# Development of a Novel Ultrasonic Spectroscopy Method for Estimation of Viscosity Change during Milk Clotting

**DOI:** 10.3390/molecules26195906

**Published:** 2021-09-29

**Authors:** István Kertész, Dávid Nagy, László Baranyai, Klára Pásztor-Huszár, Kinga Varsányi, Lien Le Phuong Nguyen, József Felföldi

**Affiliations:** 1 Institute of Food Science and Technology, Hungarian University of Agriculture and Life Sciences, 1118 Budapest, Hungary; nagy.david.szie@gmail.com (D.N.); baranyai.laszlo@uni-mate.hu (L.B.); pasztorne.huszar.klara@uni-mate.hu (K.P.-H.); k.varsanyi@campdenkht.com (K.V.); Nguyen.Le.Phuong.Lien@uni-mate.hu (L.L.P.N.); Felfoldi.Jozsef@uni-mate.hu (J.F.); 2 Institute of Biotechnology and Food Technology, Industrial University of Ho Chi Minh City, Ho Chi Minh City 700000, Vietnam

**Keywords:** ultrasonics, milk, coagulation, viscosity, spectroscopy

## Abstract

Ultrasonic testing is an emerging non-destructive testing technology with high repeatability and precision. Milk is a very complex liquid and the change of its viscosity is a highly relevant property throughout conversion into other dairy products. In the following paper, we propose a novel method for the monitoring of viscosity during enzymatic milk clotting by ultrasonic spectroscopy. An ultrasonic transducer–receiver couple with a 250 kHz nominal frequency was submerged in the samples and an enveloped sweep (“chirp”) signal was applied in a through-transmission mode. Simultaneously, the change in viscosity was measured with a rotational viscometer at a constant shearing speed. The data were analyzed with an algorithm developed by the authors for spectral ultrasonic testing. Estimations yielded a high adjusted R^2^ (0.963–0.998) and low cross-validated estimation error (RPD: 4.38–14.22), suggesting that the method is suitable for industrial use given the right instrumentation.

## 1. Introduction

Ultrasonic measurement technology developments in the last decades have focused on creating clearer images, higher resolutions [1,2,3], an increase of signal-to-noise ratios [4,5,6,7], or combinations with other non-ultrasonic techniques [8,9], for material testing and medical ultrasound tests. As the relevance and possibilities of application became evident in food production and quality control for foreign bodies [10,11], the research became focused rather on the applicability for different materials and different food matrices [12] and non-destructive monitoring of processes, including maturation [13,14,15,16,17].

El Kadi et al. [18] in 2013 published a study for estimation of optimal thawing time of frozen fish, for which they measured the acoustic parameters, using a 0.5 MHz pulser-receiver couple in a pulse-echo configuration. This study deals with phase change in time, using peak-to-peak maximum amplitude and acoustic impedance (the ability of a medium to exert attenuation on the signal). This indicates some useful features to test during phase change: liquids generally dampen the signal more than solids, and they also conduct sound waves slightly worse than solids, therefore significant changes are probable in these measures. This effect was also exploited by Aparicio et al. [19], who reported that crystallization of water can be reliably measured indirectly by the sound velocity near the freezing point. Gülseren and Coupland [20] determined the correspondence between the solution concentration and speed of sound, which might also contribute to the change in sound velocity in thawing products. This phenomenon was also observed by Rashed and Felföldi [21] in different vegetable oils, as the sound velocity strictly followed the change in temperature (R^2^ = 0.997).

Benguigui et al. [22] showed that milk clotting can be effectively measured by ultrasonic techniques, and even could distinguish between acidic and enzymic coagulation as they theorized, based on the gel formation mechanism. This was the study that sparked our interest and was the motivation to use this experiment as a basis with certain modifications. They found characteristic curves typical for enzymatic and acidic gelation while following the process with 60 MHz ultrasonic sine wave in through-transmission configuration. They measured viscosity with a rotational viscometer at 1 s^−1^ shear rate, a value at which they observed competition dynamics between aggregate formation and disintegration.

Enzymic coagulation occurs in milk as rennet enzyme is introduced in the liquid, causing casein-covered micelles to connect and form a gel matrix. The enzyme breaks the chain of the κ-casein molecules that are responsible for the micelles to repel each other, resulting in bonding of micelles. This process gradually changes the structure of the milk, turning it into a viscoelastic material, which affects the viscosity of the sample. This change in the material matrix affects the ultrasonic properties of the medium conducting the mechanical waves as the contribution of viscous and elastic features to the overall texture shift.

Studies mainly focus on the Time-of-Flight (TOF), or the acoustic attenuation parameters [23,24,25]. TOF, being the time required for the signal to travel through the material, is a single value extracted by various algorithms, which need to account for the signal form. In most cases, corrections are not necessary if the measurement setup is consistent throughout measurements. As a counterexample, a chirp signal might show different peaks at different frequencies if the signal form is not corrected for the sensitivity spectrum (most of the time not being completely uniform) of the specific experimental setup. This can be solved by choosing a signal form that is not prone to produce such problems upon processing, e.g., a short burst of constant frequency. On the other hand, different signal forms were developed primarily to exploit the different analytical features, which might be lost due to the aforementioned considerations.

Acoustic attenuation is the effect of an acoustic signal losing energy whilst traveling through media, thus the received signal has a much lower amplitude than the input signal; in effect, it describes the acoustic resistance throughout the spectrum. This descriptor has been studied extensively [23], since it is correspondent to the rheological properties of the material (a highly elastic medium is a better acoustic conductor than plastic medium) allowing scientists to draw quantitative conclusions of the rheology of a material. Acoustic attenuation is frequency-specific for a given material, meaning the testing frequency needs to be determined a priori. This is usually done by trial and error, searching for the highest correlation between attenuation and the property desired to estimate. Furthermore, undisturbed conduction of the signal to the tested sample has to be ensured, or such interferences with the measured values need to be resolved.

The issue with these two parameters lies in them being a single value. This is beneficial if we want to compare a highly descriptive feature between samples, but in many cases for highly complex materials, such as food, they do not capture a lot of the extractable information, and, from a statistics viewpoint, account for a small amount of the physical variance of the samples. It is quite understandable, therefore, why they are good descriptors for changes in less complex materials [19,20,26,27].

## 2. Results

### 2.1. Viscosity Estimation

**Figure 1:**
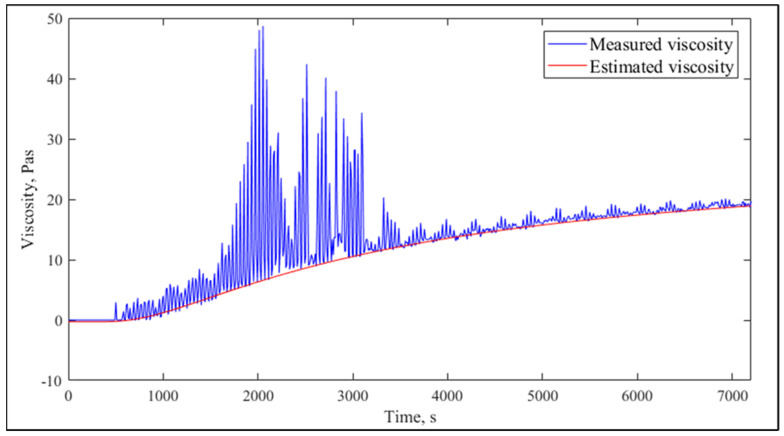
Example for measured and estimated viscosity (R^2^_adj_ = 0.9973).

The inverse negative exponential function was successfully fit to instrumentally measured viscosity curves (Figure 1). Although the fitted function slightly underestimated real viscosity values, it followed the trend well, including the lag phase. Resulting curves were similar to those reported by Benguigui et al. [22]. The adjusted coefficient of determination R^2^_adj_ was between 0.7251 and 0.9985. The highest value of R^2^_adj_ was observed for the lowest fat content (1.5%) and the lowest value for the highest fat content (3.5%). 

The adjusted coefficients of determination (R^2^_adj_) are reported in Table 1. No trend can be found in the function of fat content, but variance seems to increase. 

**Figure 2:**
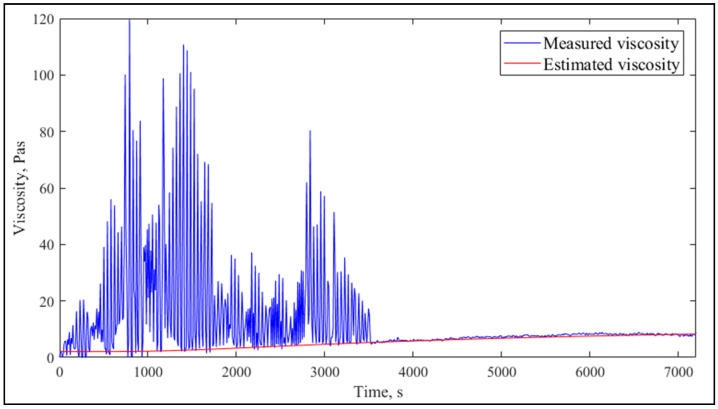
Measured and estimated viscosity with the lowest fit quality (R^2^_adj_ = 0.7251) with the lowest GOF result.

Figure 2 presents viscosity data with a fitted curve for lowest observed goodness of fit (GOF). There were numerous outliers, but the fitted function was able to follow the baseline.

### 2.2. Individual Viscosity Change

The results of the evaluation of the first set (all samples modeled individually) are shown in Table 2. with adjusted R^2^ and Residual Prediction Deviation (RPD) values included. RPD as a metric for the overall estimation descriptor was used, because it encapsulates the information regarding the uncertainty of the original dataset (as the standard deviation) as well as the prediction uncertainty (expressed as the validated root mean square error of prediction):

RPD = SD/RMSEP(1)

This makes it highly useful in characterizing the applicability of a model, or in this case, the principle behind the construction of models. As a general rule, the RPD is considered applicable for process control between 5 and 10, and outstanding at >10, meaning a very high prediction power [28].

**Figure 3:**
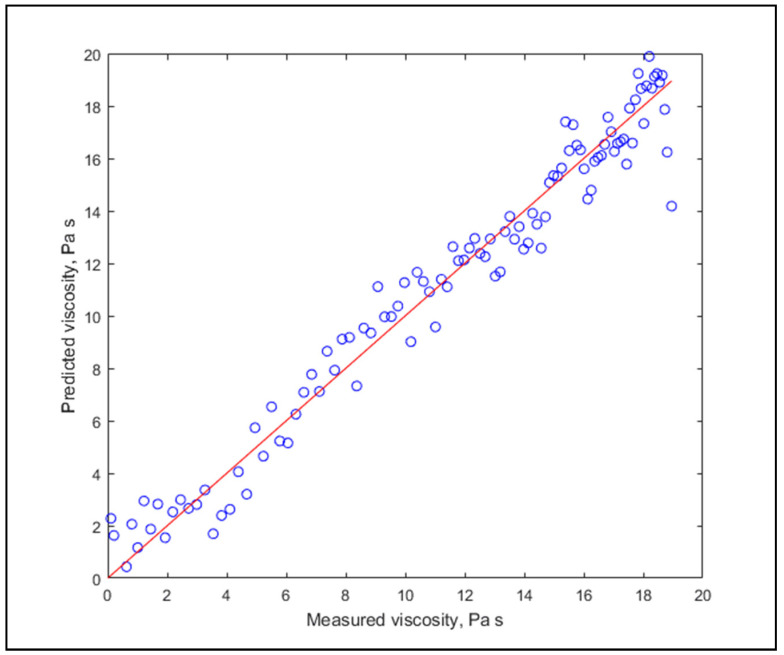
The worst prediction for individual modelling (R^2^_adj_ = 0.9632, RPD = 4.38).

**Figure 4:**
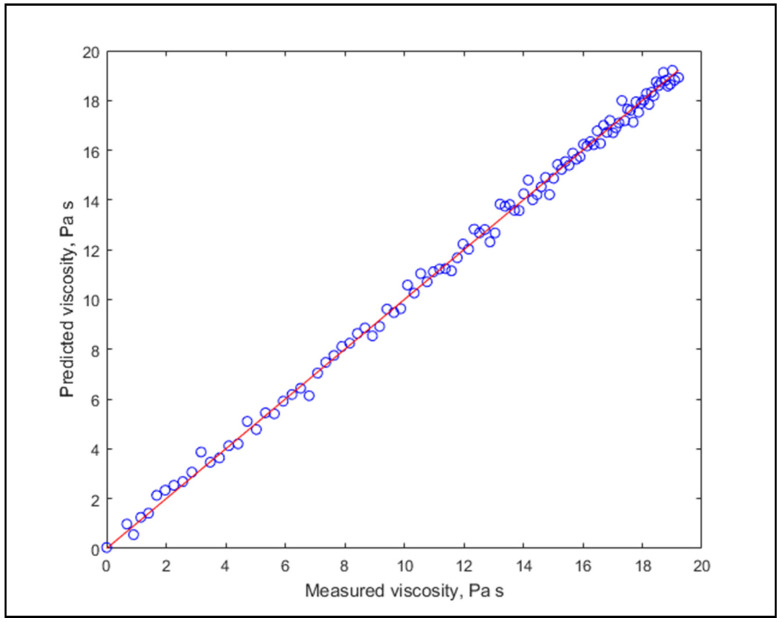
The best prediction for individual modelling (R^2^_adj_ = 0.9983, RPD = 14.22).

Goodness-of-Fit (GOF) yielded very high values, R^2^_adj_ averaging at 0.9841, and model accuracies were excellent as well, in many cases extremely high. This clearly displays the applicability of the algorithm for viscosity estimation. It needs to be mentioned that the values of RPD slightly varied with the random data partitioning of the 20-fold cross-validation, in the range of ±0.2. It might be interesting to note that there is no apparent connection between the fat content and estimation performance. Graphs of the lowest and highest estimation performances are shown in Figure 3 and Figure 4.

### 2.3. Overall Viscosity Change Estimation

In another run, all measurements were concatenated in a matrix for estimation of the corresponding viscosities. This altogether means 13 × 120 = 1560 rows to include all observations. The input variables were chosen the same way as in the previous estimation setup; this time, fat content was added as well, yielding altogether 298 variables. The second loop of Partial Least Squares (PLS) regression found the minimum of the RMSE at the 135th latent variable, which is a high, but perfectly realistic number taking into account the number of observations and that the estimator variables were already collected from an already narrowed pool of 135,168 variables, meaning 0.1% of the original figure. Furthermore, overfitting was avoided by cross-validation (this time for extra certainty; the number of folds was increased to 25).

**Figure 5:**
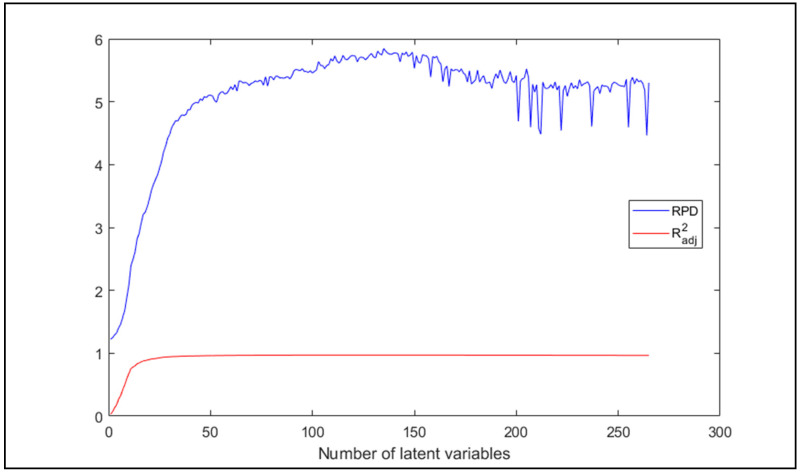
RPD and R^2^_adj_ values of prediction of all samples in the function of number of latent variables.

A total of 135 latent variables (LV) returned an R^2^_adj_ of 0.9708 and an RPD of 5.85, which means a very good, highly explanatory model with a high prediction accuracy. Following the principle of parsimony, a minimum number of LV was searched for, that still satisfies the requirements of a well-fitting model. The minimum required RPD was set to 4.00, and for R^2^_adj_, a threshold of 0.95 was assigned, which for the former was achieved at the 26th, for the latter at the 35th latent variable with 0.9514 at which the RPD value was 4.75. Their trend is shown in Figure 5.

**Figure 6:**
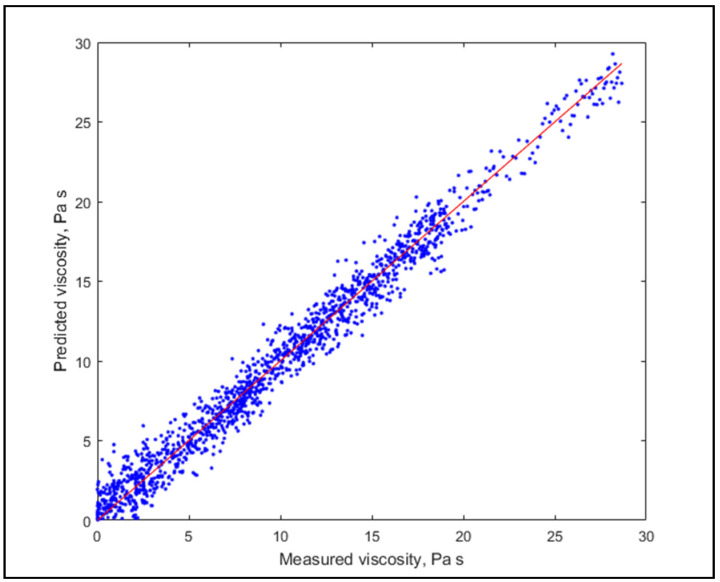
Model prediction with 135 LV.

**Figure 7:**
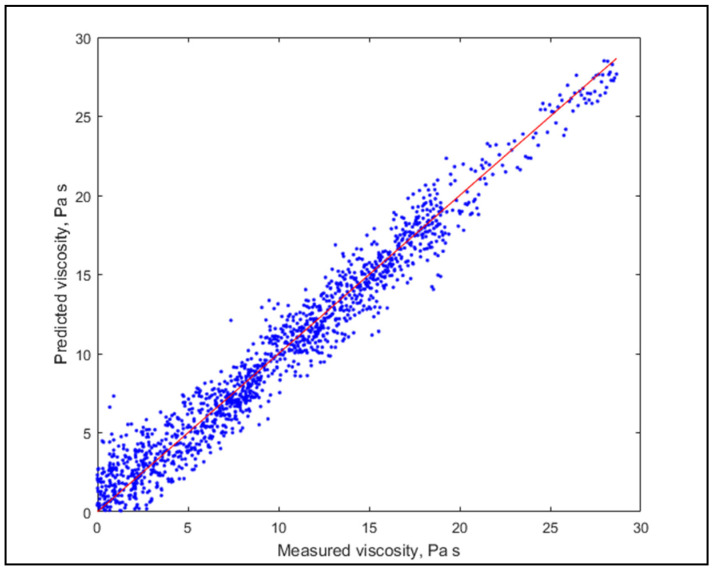
Model prediction with 35 LV.

It is visible that the two statistics increased rapidly in the beginning, but for GOF it did not improve substantially, therefore the focus should rather be on the accuracy. The estimations with 135 and 35 LV are shown in Figure 6 and Figure 7, respectively. 

Diagrams show a similarly good fit, but slightly worse prediction performance for less LV, thus (being rigorously validated) the use of a higher number of LV is suggested.

## 3. Discussion

As a conclusion, it can be asserted that the two approaches were successful in terms of prediction applicability. It has been proven that by choosing a limited number of the wavelet-transform coefficients of the ultrasonic response signal, calibration can be carried out for viscosity and therefore clotting stage estimation. Furthermore, the results suggest that the proposed post-processing method is applicable in general for predictor variable extraction for other multidomain measurements.

As most research articles in ultrasonics focus on single-value descriptors, the applicability of such metrics is limited, although in many cases sufficient. On the other hand, multivariate methods are uncommon in ultrasonics, because of the complexity of the signal and the difficulty of identifying meaningful features of the response signals.

Milk coagulation studies generally focus on measuring either the attenuation or the TOF (for sound velocity calculation) parameters at one given frequency or with impulse signals to estimate certain characteristics of coagulation. This approach resorts to the standard procedure, which has the limitation of not examining the response in a spectrum, and this requires the assumption that all relevant information can be extracted from the response of one single frequency. Although in some cases this might be true, it is certain that testing a wide spectrum will inherently serve us with more information on the material [29,30,31,32]. As Budelli et al. pointed out, measurements of only attenuation or the TOF parameter have their own specific limitations [33]. Few studies investigated the spectral response of clotting milk, and these only focused on a very limited number of testing frequencies or bandwidths, hence the same issue persists, although slightly mitigated [34,35].

Our method is generally suitable for the non-destructive, predictive determination of viscosity and therefore the state of the curdling process in enzymatic milk clotting during coagulation. The proposed novel ultrasonic spectroscopy method is suitable, applicable, and recommended for industrial use in different fields of the food industry with adaptation to the technology at hand.

The end of milk clotting can be determined by matching the specific limit values to the appropriate curd quality that is considered completed. The continuous monitoring of the milk clotting process can lead to technology optimization in small-scale (craft products) and large-scale production as well. Digital data collection and adaptive process control based on the method presented in this article leads to quality improvement of the products, capacity and yield increase, loss reduction, and more environmentally friendly production technology in the long term. The principles of our novel ultrasonic spectroscopy method can also be applied for studies in acidic coagulation in milk. In theory the use of the method can be extended for different products such as yogurt, sour cream, puddings, etc., to monitor texture properties in phase changing processes.

## 4. Materials and Methods

### 4.1. Milk Clotting Measurements

Altogether, 13 measurements were conducted, at three different milk fat contents, 1.5%, 2.8%, and 3.5%. Samples were purchased from the same manufacturer (Sole-Mizo Zrt, Budapest, Hungary), and the three different types were identical in every other feature including heat treatment (extended shelf-life, ESL). The volume of 100 mL of the samples were fortified with 0.1 g of crystalline CaCl_2_ to ensure no limitation of clotting due to low calcium content. A transducer–receiver couple was submerged in the beaker containing the milk sample, using a stand made of rigid foam to ensure a constant distance of four millimeters between the couple. The sample was heated to 38 °C and kept at this temperature for 120 min. Temperature was monitored using a Pt100 thermometer connected to a digital multimeter (VC640, Voltcraft, Duisburg, Germany). Once the sample reached the desired 38 °C, the ultrasonic measurement was started using the instruments and methods discussed in Section 4.2. Rennet (Présure simple Brun calf stomach enzyme, Alpha-Vet Kft., Budapest, Hungary) of 1000 µL was added with simultaneous agitation. A total of 5 mL of the sample was transferred for each viscosity measurement.

### 4.2. Ultrasonic Measurements

Experiments were carried out with a piezoelectric ultrasonic transducer–receiver couple (The Ultran Group, State College, PA, USA) with a nominal frequency of 250 kHz, which is the peak sensitivity frequency. To capture the input and response signals, a Velleman PCSGU250 Oscilloscope (Velleman Group, Temse, Belgium) was used and connected to a PC. The transducer was connected to the signal generator output with a coaxial cable. The software PcLab2000LT v1.12 was used to adjust signal generation and acquisition parameters: signal form and frequency, amplification and offset, signal gain, range, sampling frequency and trigger level. The acquired data were saved into ASCII text files for further processing in Matlab 2017a (The MathWorks Inc., Natick, MA, USA). Data files were generated every minute, saving 4096 data points for each measurement. Digital units were adjusted to 32 levels, resulting in 0.03125 V per unit and 0.3125 mV per unit resolution for the transducer and the receiver, respectively. In total, 120 files were generated for one experiment.

**Figure 8:**
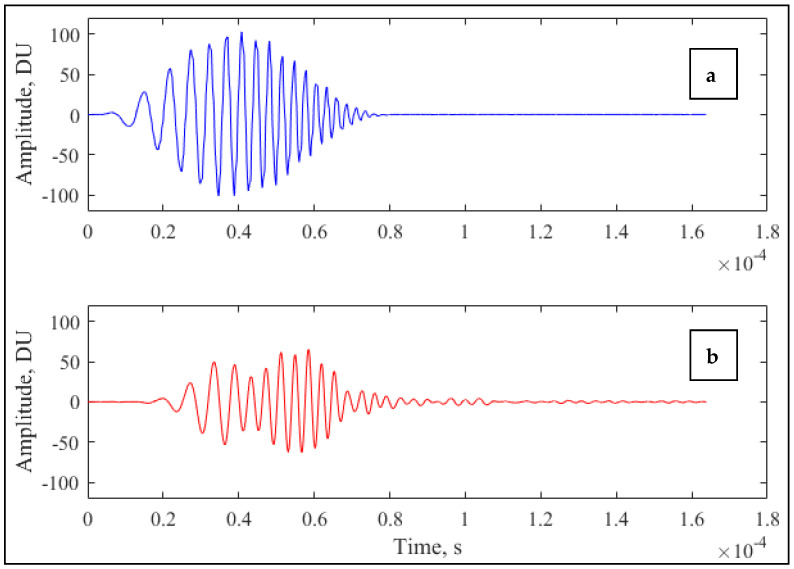
Example of “chirp” input (**a**) and response (**b**) signals.

In ultrasonic measurements, a so-called “chirp” signal was utilized. This is a double-modulated test signal with increasing frequency, enveloped by a filter. In the case of the present experiment, the applied signal had a linearly increasing frequency sweep from 50 kHz to 450 kHz and was modulated by a Hanning filter [36]. Sample input and output signals (centered to zero, denoised, unnormalized) are shown in Figure 8.

The signal sampling was set to 125 readings in 10 µs, yielding 12.5 MHz final sampling frequency, which provides sufficient resolution and data points required to analyze the generated signals. Data were analyzed using Continuous Wavelet Transform (CWT) method in Matlab 2017a software.

### 4.3. Reference Measurement of Viscosity

Measurements for the milk clotting experiments were carried out with a HAAKE RotoVisco 1 rotational viscometer (Haake Technik GmbH, Vreden, Germany), tempered at 38 °C (±0.1 °C), to match the conditions with the ultrasonic measurement setup. Viscosity was expressed in Pa·s. The measurement program was set up with an initial 10 s high speed (500 s^−1^) agitation period, followed by the 7200 s measurement with the shear rate of 2 s^−1^. Data were acquired every 10 s.

### 4.4. Viscosity Estimation in Time

A specific problem of viscosity measurement of sol-gel transformation is the formation of particles through connection of micelles, resulting in peaks on the rheogram. These peaks appear overlaid on the viscosity curve, which shows a monotonic increase in this timeframe after an initial lag phase (measuring close to zero viscosity). Focusing on the base viscosity curve, estimation results in a highly explanatory fit, henceforth estimation of the predicted viscosity values from the ultrasound signals processed with wavelet-decomposition was done.

The trend in the viscosity curves was found to follow an inverse negative exponential equation:

(2) $$\eta =A\cdot e^{-\frac{B}{t}}+C$$

*A* (Pa·s), *B* (s) and *C* (Pa·s) are the model parameters, *t* (s) is the time. This assumption is supported by the observation that the viscosity of the medium is approaching a maximum value of conversion through an enzymatic reaction; therefore, the model can be considered conclusive. The major problem of fitting is to find an algorithm that offers a generic solution for any such measurement data, meaning virtually random, non-noise induced peaks, superimposed with data following a clear trend.

The following procedure was applied: minima of the curve were calculated for consecutive, overlapping intervals of a size that ensured the absence of positive extremes caused by peaks of a specific width (2–5 data points with 0.1 Hz sampling frequency). When consecutive peaks still resulted in outliers, data were left out of analysis and curve fitting. To ensure reliability, a minimum of 10 data points were collected per interval. The sampling frequency was adjusted to 1/60 s^−1^, identical to that of the ultrasonic measurements.

### 4.5. Viscosity Estimation Based on Ultrasonic Response Signals

The main challenge of estimating viscosity lies in the fact that the patterns within the response signal can be described by the spectrum changes on the time domain using CWT. This in effect creates a hyperspectral dataset which is captured 120 times during the clotting process, resulting in 91 × 4096 = 372,736 data points as variables with 120 observations for one single run, even at a low resolution. 

The standard procedure is to extract latent variables and base estimations or classification of these, which relies on calculation of covariance matrices. Such matrix calculations for several thousands of variables are still challenging with today’s computational power. Compared to extraction of LV based on covariance, computation of variance across single variables is less demanding, therefore can be achieved in an industrially acceptable timespan. Therefore, we applied the following protocol.

The measured signals were normalized, denoised and aligned in time, therefore their absolute amplitude and latency did not affect the estimation. This ensures that only the patterns within the signal are used. The applied CWT used a Morse wavelet for all measurement signals, and the variance of the individual coefficients were calculated in time. The vector fields of the variances were calculated and the peaks of the field were identified. It was assumed that these wavelet coefficients are descriptive of the neighboring coefficients, making covariance calculation obsolete. The number of the extracted coefficients was between 7 and 32. The extracted coefficients were used as predictor variables for Partial Least Squares (PLS) regression. The optimum number of LV in the PLS model was selected according to the lowest validated RMSE value.

## Figures and Tables

**Table 1:** R^2^_adj_ values for viscosity estimations.

Fat Content (m/m%)	R^2^_adj_
1.5	0.9985
	0.9948
	0.9980
	0.9792
	0.9984
2.8	0.9862
	0.9510
	0.9973
	0.9858
3.5	0.9869
	0.7251
	0.9101
	0.9917

**Table 2:** R^2^_adj_ and RPD values of the models.

Fat Content (m/m%)	R^2^_adj_	RPD
1.5	0.9944	5.07
	0.9697	4.49
	0.9876	6.96
	0.9966	10.88
2.8	0.9688	5.20
	0.9758	4.53
	0.9983	14.22
	0.9877	6.23
3.5	0.9632	4.38
	0.9943	10.09
	0.9973	13.99
	0.9684	4.71
	0.9910	8.27

## Data Availability

The data presented in this study are available on request from the corresponding author. The data are not publicly available due to the raw data files being stored on an offline hard drive for safekeeping.

## Sample Availability

Samples are not available due to being perishable goods.
